# Measuring the Impact of Operational Stress: The Relevance of Assessing Stress-related Health Across the Deployment Cycle

**DOI:** 10.1093/milmed/usab542

**Published:** 2022-01-03

**Authors:** Niclas Wisén, Gerry Larsson, Mårten Risling, Ulf Arborelius

**Affiliations:** Department of Experimental Traumatology, Institution of Neuroscience at Karolinska Institute, Solna, Stockholm 171 65, Sweden; Department of Security, Strategy and Leadership, Defence University, Karlstad 653 40, Sweden; Department of Experimental Traumatology, Institution of Neuroscience at Karolinska Institute, Solna, Stockholm 171 65, Sweden; Department of Experimental Traumatology, Institution of Neuroscience at Karolinska Institute, Solna, Stockholm 171 65, Sweden

## Abstract

**Introduction:**

Mental health issues from intense or prolonged stress are a common concern in regard to military deployment. Deployments can objectively vary in stress exposure, but it is the individuals’ perception of that stress that affects sustainability, mental health, and combat fitness, which calls for the need of a protocol to evaluate and maintain a current estimation of stress impact. So, how can we assess the impact of stressors during different phases of deployment?

**Materials and Methods:**

We used three psychological self-rating forms, the PSS14—Perceived Stress Scale, SMBM—Shirom Melamed Burnout Measure, and KSQ—Karolinska Sleep Questionnaire, to measure the impact of stress before (T1), during (T2), and at homecoming (T3). We also wanted to see if T1 or T2 results could predict T3 results to be able to better prepare the homecoming program.The forms were handed out to Swedish soldiers deployed in Mali in 2017. The forms were collected as a way to assess the status of the mental health load at three timepoints based on the personnel function as a way to assess the current “psychological fitness level”.

**Results:**

The results show that stress measured using PSS14 was high at homecoming. The same result was observed for SMBM. No measures from T1 or T2 could however predict the T3 results.

**Conclusions:**

Taken together, we found that screening of all contingent staff is relatively easy and provides personnel with relevant data on mental health and stress at the current time. We also found that test results correlated between T1 and T2 but not with T3. This indicates that there might be different stressors that affect staff at different timepoints.

## INTRODUCTION

Military deployments are often the subject of stress studies due to their challenging nature. Deployment can take on many forms, such as combat missions, peacekeeping missions, or support missions, to name a few. It goes without saying that a combat mission in Afghanistan has the potential to be more stressful at least in regard to traumatic stress than, for example, being deployed to a logistic position in a third country where threat is limited. However, everyday stressors will occur regardless of where troops are deployed. Monitoring stress over time is relevant both to identify individuals at risk and take appropriate actions to promote health and performance for the deployment. Operational or deployment stress compared to regular everyday stress can be described as a combination of stressors that relates to the ongoing performance of a demanding task (an operation) in a military context often over long time periods. The stress load does not just origin from external stressors or demands, it is also from “wear and tear” that is a combination of load and lack of sufficient recovery over time.

Depending on perspective, operational stress can be limited to a single “operation” during a deployment or cover the whole deployment cycle. In this article, we address the relevance and importance of measuring the impact of operational stress, both at an organizational level and at an individual level.^1^ Operational stress is a term predominately used within a military context. However, it is also used with so-called first responders (police, firefighters, and paramedics) today. We can also include other categories within health care since the outbreak of the coronavirus pandemic in early 2020 has caused deployment like conditions for medical personnel globally, especially for those in intensive care units. This study, however, was conducted on military personnel before, during, and after deployment of a Swedish contingent to Mali in December 2016 to May/June in 2017. The deployment was a peacekeeping mission within the UN Multidimensional Integrated Stabilization Mission in Mali.

Military stressors during deployment cover a wide range of areas, all with their own specificity and impact on soldiers. Combat-related stressors have been shown to have the most adverse effect on mental health.^2^ Aside from combat-related stressors, there are a variety of stressors that affect deployed personnel.^3,4^ Such non-traumatic and less intense stressors during deployment have been labeled daily hassles^4^; however, military deployments do not just contain negative stressors; they also contain positive experiences, labeled uplifts.^5^ Uplifts have been shown to mitigate negative effects from other stressors.^6,7^ Regardless of the source of stress, it has to be addressed to keep the fitness level high during deployment and minimize post-deployment health issues. Organizational resources for monitoring and maintaining “combat fitness” differ between deployments due to staffing, need, resources, etc. and of cause national guidelines and regulations. There are many programs out there, such as the Deployment Health Assessment Program^8^ or Periodic Health Assessment.^9^ There are different forms assessing health at the different stages of deployment, pre-, post-, and re-assessment. It includes both an electronic questionnaire and a one on one conversation (confidential). It is an example of a structured systematic way of addressing deployment related health in all phases. In a study from 2012, Skopp et al. looked at the diagnostic efficiency of post-deployment screening. They found that the Post-Deployment Health Reassessment form had a good negative predictability, that an outcome that does NOT point toward a problem,^10^ a conclusion as important as any.

Contingents can make use of the standardized tools to assess personnel status with regard to mental status such as stress, sleep, and burnout. The use of these tools must, however, be easy to administer and have relevance for the organization. Mental health in relation to deployment is mostly related to either pre-deployment screenings such as the Periodic Health Assessment, which assess medical readiness for deployment or the post-deployment stage, i.e., what happens after coming home. During deployment, the environment is so different from normal everyday life that it is difficult to validate outcomes on self-assessments with regard to mental health; high ratings might just reflect the current stress level from operational stressors. However, it is interesting to observe if ratings before and during deployment can predict health at homecoming as measured by Shirom Melamed Burnout Measure (SMBM).

To obtain valid and relevant assessments of personnel during deployment, one cannot only rely on observation or self-reporting. Military culture is not always open for communicating stress and mental health-related issues.^11,12^ Structured and reoccurring screening can be one way to bring awareness of the accumulated load of stressors during deployment, and on the individual level it might be an indicator of when it is safe to push harder vs making sure to rest and recuperate.

Pre- and during-deployment screening can be useful tools to gain insight into the current status of troops and to maintain high performance and health at all phases, as well as homecoming screening. Together with post-deployment operation analyses, it might also bring insight into how to better prepare troops and foster resilience.

Swedish troops start pre-deployment training well in advance before being deployed. The training is based on the specific assignment the contingent will have during the deployment, and depending on ordinary profile of the regiment setting up the contingent can be more or less in line with that profile. The training period can be intensive and include novel skills training and long hours compared to regular service. It not uncommon that soldiers perceive the pre-deployment training as more stressful than the deployment itself. The only assessment of health however during the pre-deployment phase is the health examination that is aimed and giving a medical approval for deployment. During deployment no assessment or measure of troop status is obtained aside from the regular leadership. The assessment comes at homecoming where the staff must pass through a homecoming program. The program contains a medical screening, a homecoming lecture, guided group discussions, and individual screening. However for this contingent Mali 05, we tested to expanded the screening to cover the before and under phases as well, which was done as a part of the regular personnel work and was made possible by the deployment of a operational psychologist. A position that at the time was added to the contingent as a fulltime deployment.

In this study, we aim to look closer at the possible benefits of mental health assessment *screening* and its usefulness for military operational psychologists, personnel officers, and other HR-related functions such as welfare.

## RESEARCH QUESTIONS

1. Can mental health screening provide a useful tool for managing mental health in personnel deployed in military operations? Is there a relevance of making group assessment (whole contingent) to assess stress load on an organizational level, and subsequently to be better prepared for post-deployment care/follow-up.
2. Can measures from either before or during deployment predict any homecoming measures?

## METHOD

### Subjects

Data were collected from the Mali 05 Contingent in Timbuktu 2016-2017 a peacekeeping mission within the UN Multidimensional Integrated Stabilization Mission in Mali. The Swedish contingent was an Intelligence, Surveillance and Reconnaissance Task Force, with both unmanned Aerial Vehicles and reconnaissance troops. The self-rating scales were distributed and collected through the line organization and given to all active personnel regardless of rank and position. The intent when data was gathered was to get an assessment of troop status in relation to operational demands and follow-up responsibility from the armed forces that apply to all personnel. The questionnaires were anonymous; however, a small fraction of soldiers (*n =* 24) marked their id-number for all three occasions, making it possible to compare their ratings over time on an individual level using tests for repeated measures. No detailed demographics of staff were accessible from the contingent post-deployment.

### Design

Three self-rating scales were given at three time points, pre-deployment (T1), during deployment (T2), and at homecoming (T3). Scales were originally given as a part of the mental health monitoring strategy. (The mental health monitoring strategy was performed in order to identify ratings indicating risk for mental health issues within the organization at the given timepoints. The results was assessed and communicated as a part of the overall contingent assessment preparing for homecoming and follow-up.) The tests were handed out to the units for distribution and collection. Collected forms were scored and analyzed by the deployment psychologist and communicated to the Head of Personnel J1 as a measure of psychological fitness.

Statistical software used: IBM Corp. Released 2017. IBM SPSS Statistics for Mac, Version 25.0. Armonk, NY: IBM Corp.

The study was based on the forms used during the mental health monitoring strategy that was employed during this mission as enhanced strategy for mental health awareness due to the deployment of a operational psychologist, they were anonymous from the beginning so it was not possible to ask for consent to use individual data. The study was subjected to an ethical approval committee, the committee concluded the data not meet the requirements to be subject for individual consent to be used, sine no identification from data is possible Dnr: 2019-02728.

### Measures

The questionnaires used were the PSS14, *Perceived Stress Scale*, Sleep: KSQ, *Karolinska Sleep Questionnaire*, and Burnout: SMBM Questionnaire.

#### Self-rated stress

The PSS14^13^ is a 14-item self-report inventory that assesses perceived stress over the prior month. The scale covers two factors labeled positive and negative stressors. Positive stressors (seven items) address factors that are related to being able to actively control or cope with a stressor. For example: *In the last month, how often have you felt that things were going your way?* The wording of the positive items aims at assessing a sense of control or possibility to act on the subject. Negative stressors are related to perceived limited control or coping resources. For example: *In the last month, how often have you felt that you were unable to control important things in your life?* On the contrary to the positive items the wording here aims at assessing a sense of not being able to control or actively handle the stress. The sum of scores could range from 0 to 56, 0 = no stress at all and 56 = all items scored the highest value. Each item is scored on a five-level scale from 0 = never to 4 = Very often. The total score was computed by first reversing scores on positive items and then computing the sum of all 14 items. Psychometric properties of the PSS14 show a high internal consistency (Cronbach’s α) ranging from α = 0.87 to α = 0.92 over all events and groups. It shows that the items in the test to a high degree measures the same underlying aspect.  A value of > 0.80 is considered very good and > 0.90 is considered great.

#### Sleep

The KSQ^14^ consists of two parts. The first 18 items, cover four dimensions: *sleep quality, difficulties waking up, snoring*, and *sleepiness*. A psychometric evaluation and standardization of the test showed a Cronbach’s α ranging from 0.71–0.87 over all age groups and dimensions, suggesting good internal reliability.^15^ The items are rated as follows: 6, Never; 5, Seldom (few times a year); 4, Sometimes (occasionally in a month); 3, Often (1–2 times a week); 2, Mostly (3–4 times a week); 1, Always (5 times a week or more). The higher the score, the better the quality of sleep. Examples of items are as follows: *Sleep quality*, “Reoccurring waking up with difficulty going back to sleep”; *Difficulties waking up*, “A feeling of not being rested when waking up”; *Snoring*, “Apnea during sleep”; *Sleepiness*, “Involuntary sleep episodes during work”. The second part consists of seven items in which the respondent makes time estimations on subjects such as time of sleep onset and duration. Only the first part was used in this study. In the literature, KSQ scores are sometimes reversed, e.g., higher scores equal worse quality of sleep; here, however, a lower score indicates the presence of sleep disturbances.

#### Burnout

The SMBM is a widely applied self-assessment instrument assessing *burnout* through 14 items covering three subscales—physical fatigue, emotional exhaustion, and cognitive weariness. The items are rated on a scale from 1 (*Almost never*) to 7 (*Almost always*). Sample items are as follows: *physical fatigue*, I feel physically drained; *Emotional exhaustion*, I feel I am not capable of being sympathetic to coworkers and customers; *Cognitive weariness,* I feel I am not focused in my thinking. Internal consistency has been shown to be high (Cronbach’s α, 92).^16^ The higher the score the higher the risk of burnout, according to the Swedish clinical stress center in Stockholm an average of 4 indicates a possible need for professional help. In their clinic the patient average score often is around 5.^17^

## RESULTS

The response rate was approximately 40%–70% over three occasions T1–T3 (T1 = 40%, T2 = 70%, T3 = 45%). A total of 412 forms were collected (T1: *n* = 108, T2:  *n* = 184, T3: *n* = 120). Missing data were due to factors such as pre-deployment training (not all personnel at site) and operational demands during deployment, making it difficult to reach all staff at the given times. A small group of soldiers (*n =* 24) marked their forms with their id number on all three occasions making longitudinal analysis possible. The id group results was compared using *t*-test, to the results of the unmarked forms at all three timepoints. No significant differences were found for any if the tests at any of the timepoints.

### Stress

PSS14 and ANOVA of the total scores for all individuals (*n* = 289) over all timepoints (T1–T3) showed a significant result at the *P *< 0.05 level for the three conditions (F(2, 286) = 4.52, *P* =0.012). *Post hoc* Tukey HSD tests showed that the main change was between T2 and T3. An ANOVA of the positive subscale found no significant change, while an ANOVA targeting the negative subscale showed a significant change over time at the *P *< 0.05 level. (F(2,286) = 8,33, *P* = < 0.001). *Post hoc* Tukey HSD tests showed that the difference was between T2 and T3 (Table I.).

**TABLE I.** ANOVA PSS14 Sum, Negative Subscale NS and Positive Subscale PS with Post Hoc Test Tukey HSD

					Tukey’s HSD Comparisons *P*-values	
Time	Scale	*n*	Mean	*SD*	T1	T2
T1	Sum	107	18.57	7.68		
T2	Sum	112	16.65	6.97	0.128	
T3	Sum	70	19.89	7.18	0.470	0.011
T1	NS	107	10.45	4.28		
T2	NS	112	9.21	4.27	0.082	
T3	NS	70	11.84	4.16	0.085	<0.001
T1	PS	107	8.33	3.96	–	–
T2	PS	112	7.42	3.78	–	–
T3	PS	70	8.04	3.65	–	–

### Sleep

The KSQ showed no change over time in either the total sum or any of the subscales. Total sum were T1 (*m *= 90.3, sd 10.6), T2 (*m = *90.4, sd 10.3), and T3 (*m = *90.3, sd 10.3).

### Burnout

The SMBM total score showed a small but significant increase over time using ANOVA (*F*(2, 378) = 3.6, *P* = 0.028). *Post hoc* Tukey’s HSD test showed that the main change was between T2 and T3 (Table II).

**TABLE II.** ANOVA SMBM, with Post Hoc Test Tukey HSD

				Tukey’s HSD Comparisons *P*-values	
Time	*n*	Mean	*SD*	T1	T2
T1	105	28.14	12.49		
T2	160	27.89	12.62	0.987	
T3	116	31.89	14.02	0.084	0.033

**FIGURE 1.**
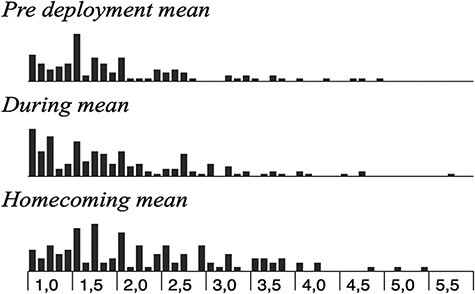
Distribution of individual mean on SMBM over T1–T3.

The mean score at the group level was well below the clinical level at all times (an item score of 4 indicates a clinical level). There are, however, some individuals with a mean above 4, at T1, there are five individuals (*n* = 105), at T2, there are seven individuals (*n* = 160), and at T3, there are seven individuals (Figure 1).

#### Longitudinal measures

The correlation matrix (Table III) shows that the test results correlate mainly within each test event (T1–T3). The correlations, however, are not so high that they indicate that the tests measure the same underlying factors. Notably, there was basically no correlation between T1 or T2 with T3, and there was only a slight correlation of a lower sleep quality score that is less than 0.45, the lower limit for moderate correlation, between T2 and T3 (lower is worse sleep quality, hence the negative correlation). There is however moderate correlations between PSS-14, KSQ, and SMBM at homecoming.

**TABLE III.** Correlation Matrix for All Sums and PSS 14 Subscales (*P* and *N*) at T1–T3

		Baseline					Under deployment					Homecoming				
		PSS-14	PSS-14p	PSS-14 n	KSQ	SMBM	PSS-14	PSS-14p	PSS-14 n	KSQ	SMBM	PSS-14	PSS-14p	PSS-14 n	KSQ	SMBM
Baseline	PSS-14															
	PSS-14p	0.88^b^														
	PSS-14n	0.86^b^	0.66^a^													
	KSQ	−0.38	−0.29	−0.38												
	SMBM	0.55^a^	0.46^a^	0.53^a^	−0.49											
Under deployment	PSS-14	0.47^a^	0.38	0.54^a^	−0.28	0.41										
	PSS-14p	0.43	0.45^a^	0.37	−0.17	0.29	0.84^b^									
	PSS-14n	0.33	0.18	0.51^a^	−0.31	0.38	0.66^a^	0.21								
	KSQ	−0.35	−0.31	−0.46^a^	0.48^a^	−0.23	−0.40	−0.27	−0.33							
	SMBM	−29	0.17	0.49^a^	−0.35	0.53^a^	0.52^a^	−0.32	0.50^a^	−0.51^a^						
Homecoming	PSS-14	−0.10	−0.17	−0.05	−0.02	−0.06	0.02	0.05	0.03	0.03	0.11					
	PSS-14p	−0.18	−0.23	−0.11	0.03	−0.10	−0.02	0.06	−0.06	−0.02	−0.04	0.88^b^				
	PSS-14n	−0.04	−0.10	−0.01	−0.06	−0.01	0.04	0	0.06	0.05	−0.17	0.92^b^	0.65^a^			
	KSQ	−0.02	0.02	0.06	0.08	−0.08	−0.38	−0.24	−0.42	0.24	−0.30	−0.53^a^	−0.36	−0.60^a^		
	SMBM	−0.06	0.12	−0.04	0.02	0.02	0.01	−0.08	0.08	−0.05	0.02	0.71^a^	0.55^a^	0.70^a^	−0.62^a^	

Moderate correlations 0.45–0.75 are marked with ^a^.

Strong correlations >0.75 are marked with ^b^.

## DISCUSSION

We asked if mental health screening could provide a useful tool for managing personnel health during military operations, and if there is relevance for making a group assessment (whole contingent) to assess the stress load of the organization to be better prepared for post-deployment care and follow-up. We also asked if such a screening could predict individuals at risk during homecoming.

The results showed only small fluctuations in values over time for sleep, stress, or burnout, the results indicate that there was no significant impact of stress during this particular deployment. A clinical relevant value on SMBM is a mean score of 4.4 or higher^18^ and at homecoming there were seven individuals who scored a result over 4. The PSS14is not a diagnostic tool in that it has a given cut off, it is “a perceived stress measure”. It is a relevant tool for follow ups of stress exposure since you get a baseline measure to refer to (e.g., has stress increased or decreased). The study would have benefitted from data being repeated measure’s e.g., tied to individuals over time. The unidentified nature of the data only allowed for group comparison except for a smaller group that marked their forms. However, the results did not differ between the soldiers that marked their forms compared to those who did not indicating that the group comparison is a valid measure.

The lack of change over time might be attributed to the specific situation, e.g., the stress and workload of the particular deployment in combination with the leadership and pre-existing psychosocial culture that, if positive, can work as a buffer towards stressors,^19^ and if negative might lower the tolerance at both the individual and group levels.^20^ Regardless of whether the results differ over time, the results provide an estimate of the status at the time of assessment. In this case, we can infer that the troop scores indicated high psychological fitness over all tests at all test events. Pre-deployment scoring showed good readiness to deploy and indicated no areas of worry. During deployment, the results indicated a small but positive effect that is consistent with previous studies of Swedish peacekeeping troops in Afghanistan,^21^ that is, a slightly lower score on PSS14 during deployment compared to pre- and post-deployment scores. Neither KSQ nor SMBM burnout measure results indicated any alarming scores during any of the timepoints. The results from the sample events give a valid “temperature” of the psychological fitness level of the troops. As argued in the introduction, the results can be used by deployment psychologists and personnel to convey a valid assessment of the troops during relevant phases during the deployment cycle. In a deployment environment, functions such as personnel and welfare have an important role of providing support to the command and the soldiers, and picking up on signals from screening forms might give a more valid assessment of the current status than what will surface with just regular leadership. We chose to use anonymous forms, although some marked the forms with id. The assumption that anonymous forms provides more accurate data might be an assumption to challenge, as there are other options such as confidential forms (access results limited to the provider of support)^22^ the best alternative might be depending on the culture of the organization at hand.

The results, a slight increase in both PSS 14 and SMBM between T2 and T3. In this particular deployment, might depend on that there was a change in the immediate risk during the last weeks leading up to homecoming. Several attacks on the camp with grenade launcher resulting in casualties on the supercamp (not the Swedish contingent) and one injured Swedish soldier (shrapnel) increased the stress of many of the soldiers. The results at homecoming might be a reflection of that intensification. Aside from that, coming home is a significant change for most (ref homecoming stress) and that by itself can account for some of the higher scores. For some, it is an easy transition; however, for others it is harder leaving their group and the close relations that many form during deployment.^23,24^ Some individuals have a harder time readjusting, and due to differences in mental health impacts from deployment, there are different needs of follow-up.^25–27^ By assessing the whole, or at least the majority, of the contingent during deployment, we will have a pre-homecoming assessment reflecting on the psychological load perceived by the soldiers. In this case, the design did not provide an adaptable homecoming program based on the results, both due to attacks coming after the T2 assessment and since the homecoming questionnaires were collected during homecoming. To capture the effects of the attacks and minimize the impact of transitioning home, assessments should have been given prior to homecoming. Neither did the result from T1 and T2 provide any predictive capacity for T3 home arrival, as argued, which could have been different if another assessment had been given after the attacks, given that stress and mental health assessments are dynamic and respond to environmental stressors. After a significant and potentially traumatic event, one should regard the previous assessment as outdated. However, the question was not limited to this contingent, and the focus was on the usability of the method itself.

## CONCLUSION

A set of selected forms that address stress-related issues and risk at several time points before, during, and after deployment will provide information that can make a difference in regard to identifying significant impacts in troops. Anonymity allows for honesty while providing one position number can be optional and a way to signal for support if needed. Combat fitness can be seen a composite construct where psychological factors account for a significant part as well as physical fitness. Relying on gut feeling to assess stress is not sufficient; we therefore tested one way to assess the whole contingent. Even though there were missing data due to operational demands, we still obtained enough data to provide the command with data concerning psychological status. We conclude that this way of assessing the troops is a valid contribution in a potentially high stress environment. The next step is to use digital platforms instead of paper and pen, minimizing time spent transferring data before analysis. There are many recourses spent on follow-up programs, veteran mental health support, etc. Simply assessing stress at several times over the deployment phases will not changes that need when it comes to traumatic stress. Still cumulative stress and stress coming from non-combat stressors can be assessed, and if identified, these should be mitigated before wear and tear sets in. We need to take the guesswork out of troop assessment and apply psychological measures not just to diagnose those in need but to be proactive and adapt to sudden changes in combat fitness due to trauma or wear and tear.
